# How People With Vision Impairment Use Their Gaze to Hit a Ball

**DOI:** 10.1167/tvst.14.1.1

**Published:** 2025-01-02

**Authors:** Ward Nieboer, Carin M. Svensen, Kjell van Paridon, Debbie Van Biesen, David L. Mann

**Affiliations:** 1 International Paralympic Committee, Bonn, Germany; 2 Vrije Universiteit Amsterdam, Department of Human Movement Sciences, Amsterdam Movement Sciences and Institute Brain and Behaviour Amsterdam (iBBA), Amsterdam, the Netherlands; 3 KU Leuven, Faculty of Movement and Rehabilitation Sciences, Department of Rehabilitation Sciences, Leuven, Belgium; 4 Anglia Ruskin University, Faculty of Science and Engineering, Department of Psychology and Sport Science, Cambridge, United Kingdom

**Keywords:** gaze behavior, vision impairment, eye tracking, tennis, dynamic targets

## Abstract

**Purpose:**

Understanding the impact of vision impairment on dynamic tasks requiring visual processing is crucial for developing effective adaptive strategies that support individuals with vision impairment in optimizing their performance in natural tasks. This study aimed to establish the gaze patterns used by individuals with vision impairment when hitting a moving target.

**Methods:**

Nineteen tennis players with vision impairment were recruited and their eye and head movements were tracked while they returned tennis serves.

**Results:**

Participants used a variety of different strategies to track the ball visually, dictated largely by the nature of their impairment rather than its severity. Cluster analysis showed distinct strategies based on the type of vision impairment: those with peripheral vision loss foveated the ball closely and avoided predictive eye movements; those with poor oculomotor control initially tracked the ball but lagged as it approached; and those with central vision loss used a variety of strategies that did not align with the use of a single preferred retinal locus: some tracked the ball using a single preferred location in their peripheral vision, some switched the area of retina used to track the ball, and another did not move their gaze at all.

**Conclusions:**

Tennis players with vision impairment adopt a variety of impairment-specific adaptations to their gaze-tracking strategies, enabling them to successfully hit an approaching tennis ball despite severe vision impairments.

**Translational Relevance:**

This study provides insight into the impairment-specific gaze strategies that well-adapted individuals with vision impairment adopt when hitting a moving target.

## Introduction

In interceptive ball sports such as tennis, cricket, or baseball, in which a player needs to determine the path of the ball and the timing of contact with it, the limits of the player's visual-motor system can be reached.^1^ Owing to the limited time available to process a fast-moving ball, players must make predictive judgments about its trajectory.^2^^–^^5^ Gaze tracking helps to reveal how athletes adapt their gaze behavior to track and make predictions about the future location of a fast-approaching target such as a ball.

Previous studies have described how athletes without vision impairment adapt their gaze behavior to allow them to intercept fast-approaching targets, for instance in tennis,^5^ cricket,^4^^,^^6^^,^^7^ and baseball.^3^ From ball release, athletes generally initially use a combination of eye and head movements to engage in the smooth pursuit of the ball, tracking the moving ball foveally for the first 50% to 80% of the ball trajectory to maintain accurate visual information.^3^^,^^4^^,^^6^ Athletes then often transition from pursuit to perform an anticipatory eye movement, where they direct their gaze—by means of a predictive saccade—to a specific location ahead of the ball's trajectory.^8^ To intercept a bouncing ball, a predictive saccade typically occurs just before the ball bounce, regardless of its speed.^8^ Predictive saccades are typically directed a few degrees above where the ball will bounce; soon after the ball bounce, vertical eye movements realign gaze with the ball.^2^^,^^5^ The predictive eye movements are not just a result of predicting the future location of the ball based on its current trajectory; rather, they are produced using complex experienced-based models that rely on prior knowledge of the elasticity of the ball and how it is affected by gravity.^2^ Following bounce, some athletes attempt to again track the ball foveally, whereas others attempt another predictive saccade to align gaze with the future location of where the ball will be intercepted at the moment of ball contact.^6^ In sports where the ball does not bounce (e.g., baseball), some batters do also attempt a saccade forward to the contact point.^3^ In essence, these findings emphasize the high level of visual-motor coordination needed to acquire visual information through a complex coordination of the smooth tracking of a fast-moving ball in combination with anticipatory gaze strategies that predict the future ball trajectory and time of arrival.

It might be reasonable to assume that individuals with vision impairment would struggle to intercept a fast-moving target such as a tennis or cricket ball given the visual demands inherent of the task. However, those with vision impairment, through adaptive/Para sports, engage in competitive ball sports such as cricket, baseball, and tennis. Sport adaptations are often put in place to make the sport more accessible for those with impairment—for example, by enhancing audio feedback (e.g., a ball with sound). Despite these adaptations, evidence has shown that athletes who are partially sighted still rely on vision and therefore perform better than functionally blind athletes in, for example, judo,^9^ swimming,^10^ alpine skiing,^11^ and football^12^ (where functionally blind, in blind sports, is typically those with acuity worse than 2.6 logarithm of the minimum angle of resolution [logMAR]). In tennis for athletes with vision impairment, athletes with residual vision seem to perform better than those who are fully blind and therefore must possess some form of ability to use their limited visual capabilities to pick-up information about the ball flight. It remains unclear, though, how they use their limited vision and what it is they are relying on to facilitate their performance. The knowledge gained from studying such interception tasks can be useful in providing a fundamental understanding of how vision is used by people with vision impairment more generally. Moreover, information gained from this well-learned task can provide vital insights into the optimal strategies when performing challenging visual-motor tasks in the presence of vision impairment. Understanding these strategies is crucial not only for providing insight into how individuals with vision impairment adapt to dynamic sports tasks, but also for informing targeted interventions and training methods. Such knowledge can help to optimize performance in sports and daily activities, contributing to enhanced rehabilitation programs for those with visual impairment.

There are likely to be a range of different gaze strategies that individuals with vision impairment could use when intercepting a ball, with those strategies likely to depend on the type of impairment those persons have. For example, athletes with central vision loss may attempt to track the ball with their remaining peripheral vision. Peripheral tracking could be achieved by maintaining the ball in their preferred retinal locus,^13^^–^^15^ that is, the area in the remaining retina that affords the best acuity, or perhaps even that is most sensitive to velocity.^16^ Alternatively, it could be that it is too challenging or even disadvantageous to rely on the preferred retinal locus and so a different strategy might be necessary, for instance, using a combination of different parts of the retina, or by not moving the eyes at all. Conversely, athletes with peripheral vision loss may avoid any predictive eye movements and instead prioritize close foveal tracking of the ball to prevent losing sight of it in the periphery. Little is known about the commonalities and dissimilarities in gaze patterns between athletes with vision impairment during interception.

There is compelling evidence from fundamental laboratory studies that might provide clues about what would be an optimal strategy for individuals with vision impairment to adopt when tracking a moving target. For example, studies investigating smooth pursuit performance in individuals with vision impairment, such as those with macular degeneration or amblyopia, have demonstrated deficiencies in eye velocity matching (gain) compared with healthy controls.^17^^–^^20^ These conditions often result in central vision loss or impaired binocular coordination, which are assumed to impair the ability to track moving targets smoothly. For instance, individuals with macular degeneration have reduced visual acuity and may rely on peripheral vision, which may lack the sensitivity required for precise tracking of fast-moving objects like a tennis ball.^20^ Moreover, impaired binocular coordination in conditions such as amblyopia can affect motion perception and visual processing speed.^19^^,^^21^^,^^22^ However, the specific impact of poor smooth pursuit performance on the ability to accurately track a tennis ball during its trajectory in real-world sports scenarios remains an area requiring further investigation, particularly considering that much of the ball tracking is performed by movements of the head rather than the eyes.^6^^,^^16^ For instance, Mann et al. (2013)^6^ demonstrated that cricket batters rotate their head in such a way that the ball remains in a consistent direction relative to the head throughout the ball's approach. In other words, if they kept their eyes still and just moved their head as they do, then their gaze would be directed toward the ball throughout most of its trajectory.^6^ Such a strategy might be particularly helpful if moving the eyes appropriately (e.g., smooth pursuit eye movements) is problematic.

Prior research also provides an indication about how gaze behavior might be altered as a result of peripheral visual field loss during ball tracking (e.g., due to glaucoma and retinitis pigmentosa).^23^^–^^26^ Individuals with retinitis pigmentosa have been shown to display gaze behavior that is significantly less dispersed when compared with healthy controls, suggesting that peripheral field loss leads to less eye movements toward their blind areas^24^^,^^26^ (see also Ryu et al., 2013, 2015^27^^,^^28^ for examples with simulated peripheral loss). In individuals with glaucoma, when searching for an object in a crowded scene or among a set of distractors, visual field loss results in longer search times and fewer saccades toward blind areas.^23^^,^^25^ Conversely, some studies show that those with peripheral vision loss in some situations prefer to perform more eye movements, particularly as a deliberate strategy to find potential objects of interest in case they are lying within a peripheral area of blindness.^29^^–^^31^ It does seem reasonable to assume though that individuals with peripheral visual field loss, in a ball tracking task, should be expected to track the ball very closely, and to avoid predictive saccades that are potentially toward blind areas, given that the ball is the most pertinent source of information in a hitting task.

The aim of this study was to uncover the gaze patterns used by individuals with vision impairment when hitting a moving ball. Specifically, the research sought to uncover how individuals with different ocular conditions and varying degrees of vision impairment compared in their ability to visually track the ball and predict its trajectory. We recruited active tennis players with vision impairment who could be classified into one of four groups (B4, B3, B2, or B1) based on their level of central and/or peripheral vision loss (as measured by visual acuity and visual field). We expected athletes with central vision loss to have a limited ability to track the ball smoothly, resulting in considerable variability in the angle between gaze and the ball during ball flight. Conversely, we expected that those with peripheral vision loss would demonstrate tight ball pursuit to prevent losing the ball in their periphery. The results were expected to provide fundamental insights into how those with vision impairment make use of their remaining vision to achieve interception, and from an applied perspective were expected to help inform training and rehabilitation programs to assist individuals with impairment to enhance their functioning in everyday tasks outside of the clinic.

## Methods

### Participants

Nineteen competitive tennis players with vision impairment were recruited for this study (age 46.1 ± 15.1 years; 9 females). Vision impairment was defined as having reduced or no vision caused by damage to the eye structure, optical nerves or pathways, or visual cortex of the brain (International Blind Tennis Association, n.d.). Players were recruited from tennis clubs in England. There were no specific exclusion criteria based on the type of ocular condition, ensuring a diverse representation of participants with various vision impairments. The study was approved by the ethical board of the KU Leuven and was conducted in accordance with the tenets of the Declaration of Helsinki with the informed consent of all participants.

During competition, tennis players with vision impairment are grouped into classes based on their performance on tests of visual acuity and/or visual field. There are four classes in VI tennis: B4, B3, B2, and B1, with B4 consisting of the least severely impaired and B1 the most severely impaired players (International Blind Tennis Association, n.d.). The B4 class consists of players with a visual acuity ranging from 0.5 to 0.9 logMAR (inclusive) or a visual field of less than 40° in diameter. The B3 class comprises players with a visual acuity ranging from 1.0 to 1.4 logMAR or those with a visual field of less than 10° in diameter. The B2 class includes players with a visual acuity ranging from 1.5 to 2.6 logMAR. Players in the B1 class, characterized by visual acuity worse than 2.6 logMAR, were excluded from the study because they are required to wear eyeshades during play and therefore eye tracking was not possible (International Blind Tennis Association, n.d.). For this study, three participants belonged to the B4 class, eight to the B3 class, and seven to the B2 class. The class allocation provided some information on the severity of impairment of the included participants.

### Experimental Setup

The experiment took place on a tennis court adapted for VI tennis, measuring 18.28 m in length (regular court, 23.8 m) and 8.23 meters in width (regular court, 10.9 m). The service line was positioned 2.74 m from the baseline (regular court, 5.4 m) and, identical to a regular court, 6.4 m from the net, with the net height set at 90 cm. Participants used a ball specially designed for VI tennis, which is larger and softer compared with a regular tennis ball. Additionally, VI tennis balls have ball bearings within the ball to provide acoustic information during play. The sound emitted from the ball supplements the reduced visual information available to the players, this is a required adaptation to the sport (Blind Tennis Association, n.d.). The tennis rackets used had heads with a maximum diameter of 63.5 cm.

Similar to the rules of the International Blind Tennis Association, players were allowed multiple bounces before the ball was hit, with a maximum of three bounces. Participants were encouraged to use as minimal number of bounces as possible.

Two experienced sighted tennis players, both with more than 10 years of tennis experience (mean = 13 years of tennis experience), served the balls during the experiment. Each participant only faced one of the two servers. Servers were required to deliver the serves as second serves, using approximately 75% of their power, and to apply a consistent type and amount of spin to ensure consistent serve conditions, as recommended by Sáenz-Moncaleano et al. (2018).^32^ Although we were strict on the serve speed and spin, the depth of the serve—that is, the distance of the ball bounce from the participant—was not explicitly controlled as long as it bounced within the service box following the rules of the game. The median serve speed was 52.1 km/h (interquartile range [IQR], 50.4–55.2 km/h). To calculate the serve speed, the distance the ball travelled was calculated for 10 trials per participant by determining where the ball bounced from the scene camera footage and determining how far this was from the net or service line, subsequently adding this number to the length of a half court (9.14 m). The travelled distance was divided by the time from serve to ball bounce to calculate the serve speed. The median serve speed for server 1, who served only to participant 1, was 59.6 km/h (IQR, 55.8–60.2 km/h); this was significantly faster (Welch t = 6.05; *P* < 0.001) than for server 2, who served to the remainder of the participants with a median speed of 51.7 km/h (IQR, 50.1–53.1 km/h). The difference in ball speed was considered in the data analysis (see Data Analysis).

Participants’ gaze was recorded when returning serves using the SMI iViewETG eye-tracking glasses (SensoMotoric Instruments GmbH, Tetlow, Germany). The eye and scene cameras of the glasses had a sample frequency of 30 Hz. The eye-tracker used in this study operates at a relatively low sampling rate (30 Hz), which means that relatively larger saccades can be detected, but that it might not be possible to reliably analyze the dynamics of those saccades (e.g., velocity or acceleration) and that some small saccades particularly in early ball flight might be missed.^33^ The scene camera had a resolution of 1280 × 720 pixels and a field of view of 60° horizontally and 46° vertically. The manufacturer reports that gaze position accuracy is within 0.5°. The data were recorded using iViewETG (Version 1.0) recording software, which was running on a small laptop (Lenovo X220, ThinkPad, IBM, Armonk, NY) securely placed within a waist pouch tightly wrapped around the lower back of each participant. Calibration was performed, after fitting the glasses, using a three-point calibration procedure. This involved using three colored pieces of paper (colors: light green, pink, and yellow; 86 cm × 61 cm) with a black square printed on the middle of the paper (41.5 cm × 29 cm) affixed to a wall and viewed from approximately 5 m. Participants were instructed to look at the black squares one by one to calibrate the eye tracker. Participants with central vision loss are, in our experience, generally very good at directing their affected fovea toward a target. Therefore, we performed our analyses assuming that the gaze direction established during calibration represented the direction of the fovea, even in those with central vision loss. Accordingly, gaze is generally directed away from the ball at the moment of racket-ball contact for those with central vision loss (e.g., participants 10, 13, 14, 15, and 17). Calibration checks were conducted halfway through the experiment for each participant to ensure data quality and minimize drift.

In addition to the eye tracking, two high-speed cameras (GoPro Hero4; GoPro, San Mateo, CA) were positioned beside the net, with each camera recording one side of the court. This setup allowed confirmation of the moments of racket-ball contact by the server and receiver, and of ball bounce if any of those events were not clearly visible in the footage captured by the eye-tracker's scene camera.

### Procedure

The experiment began with a 10-minute tennis-specific warm-up session, during which participants engaged in their own warm-up exercises with their peers. After the warm-up, participants were equipped with the eye-tracking glasses and a waist pouch containing the computer for data recording.

To ensure familiarity with the equipment, participants were given the opportunity to practice returning serves with the eye-tracking glasses and waist pouch as much as needed before the actual testing commenced.

The test procedure consisted of a total of 40 serves per participant, divided into 4 blocks of 10 serves each. In each block, the server, standing diagonally opposite of the serve direction, directed the serves to different locations within the service boxes, specifically “wide advantage,” “middle advantage,” “wide deuce,” and “middle deuce.” Balls served to the wide advantage location were directed to the left side of the court from the receiver's perspective and to the left side of the service box (toward the side line); middle advantage balls were directed to the left side of the court, but on the right side of the service box (toward the middle line). Similarly for the wide and middle deuce, where deuce means to the right side of the court. Participants were instructed to return the serve as they would in a competitive game. The order of serve direction, wide or middle, and advantage or deuce, for each block of 10 serves, was determined randomly for each participant. The participants were given a break between blocks for as long as they needed.

Replicating actual VI tennis games, the service motion began with the server calling “Ready?” and waiting for the receiver to respond with “Yes.” Immediately before hitting the ball, the server shouted “Play.” This standard procedure in VI tennis ensured that the receiver had auditory information about the server's location on the court. In cases where the server failed to place the ball within the intended service box, the trial was redone. Trials where the ball was hit, regardless of how successful the return was, were considered for analysis.

### Data Analysis

We used the SMI BeGaze analysis software (SensoMotoric Instruments GmbH, Version 3.4) to analyze the gaze data. We extracted both the scene camera videos and gaze location for each trial using the BeGaze software. A trial was defined as the time interval from the server calling “Ready” until the racket-ball contact of the receiver.

**Figure 1.**
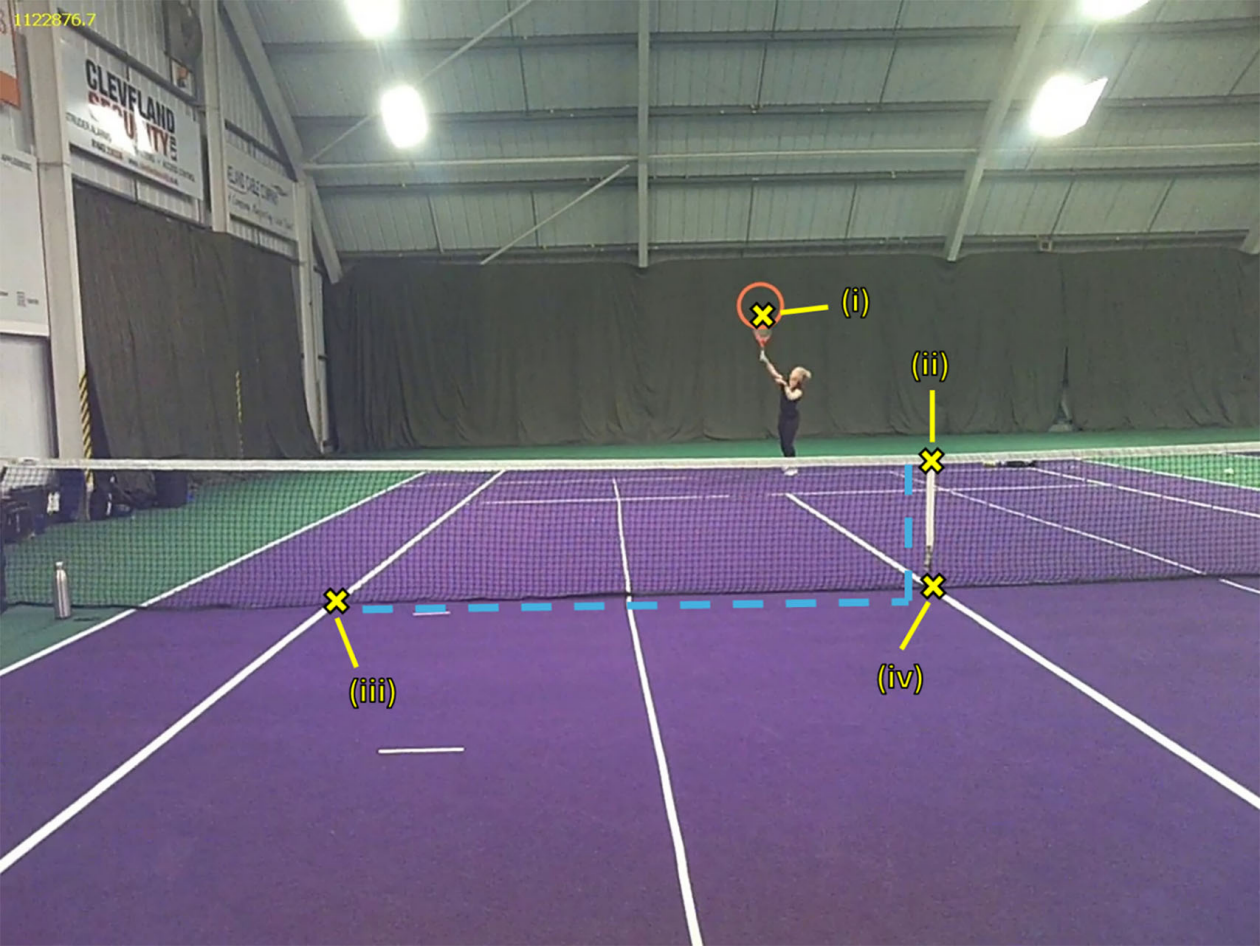
The spatial locations that were manually identified in each frame: (i) ball location, (ii) top of the net at the midline, (iii) bottom of the net at midline, and (iv) intersection between sideline and net at receiver side.

We extracted video frames from the scene camera footage using the OpenCV library in Python (Version 3.9). Using the same open-source library, for each extracted frame, four different spatial locations were manually identified and digitized: the (i) ball, (ii) top of the net at the midline, (iii) bottom of the net at midline, and (iv) intersection between sideline and net at receiver side (Fig. 1). Gaze location was already available in screen coordinates from the eye tracker software. The gaze data were aligned with the corresponding frames based on timestamps.

**Figure 2.**
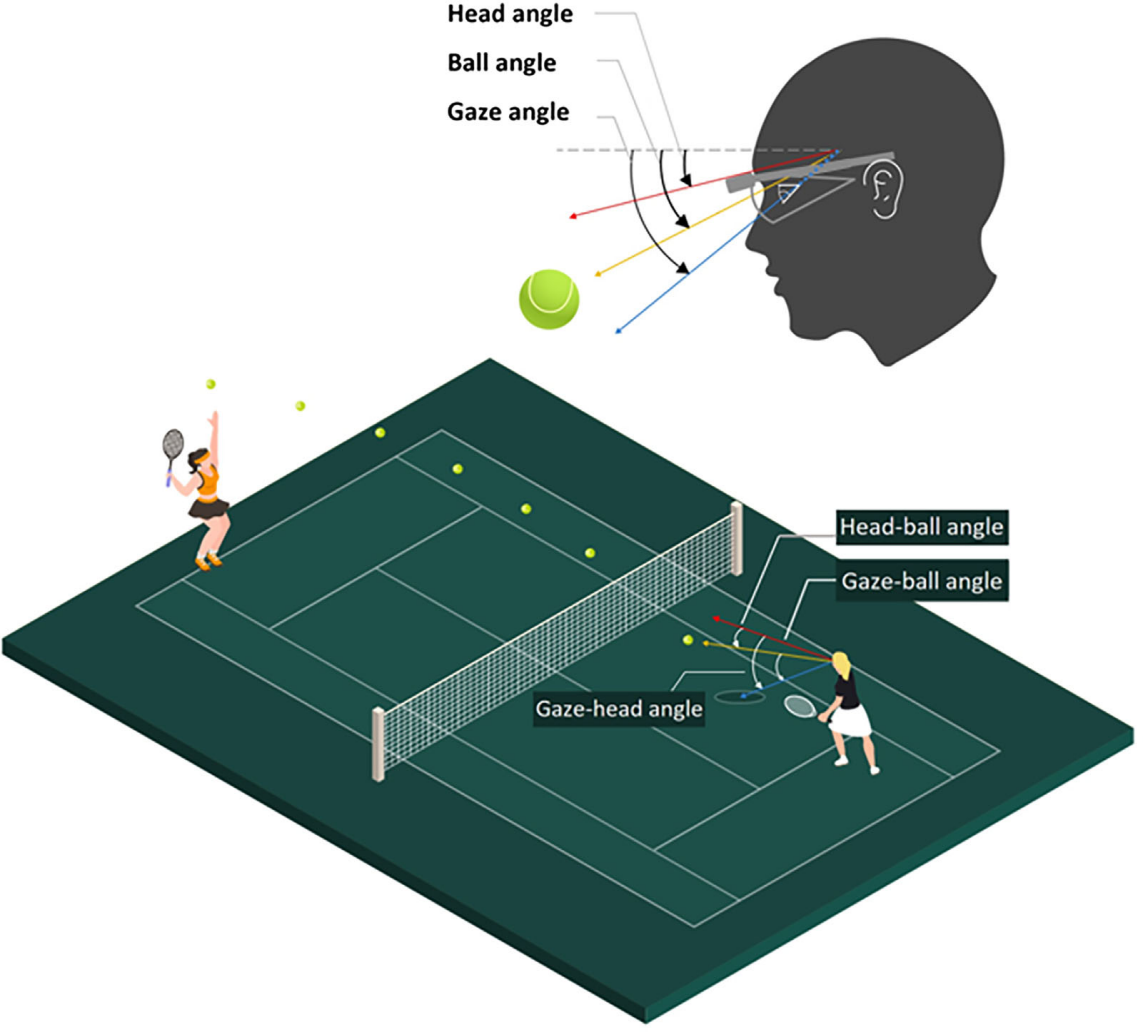
(Top) Head (red), ball (yellow), and gaze (solid blue line, dotted after eye's rotation center) angle (in degrees) at the eye, with the vertex (angular point) located at the eye's center of rotation, relative to the initial direction of ball release (dotted line). (Bottom) The resulting relative angles that represent the movement of the eyes and head relative to the ball and the eyes relative to the head.

The location of the ball, gaze, and top of the net were used to calculate three raw angles (in degrees) subtended at the eye (with the vertex being the eye's center of rotation) at the moment of the server hit: the ball angle, gaze angle, and head angle (Fig. 2). The final two reference points (bottom of the net and baseline) were used to calculate and correct for head rotation to ensure that all three angles were reported relative to global rather than local coordinates. Three relative angles were computed to illustrate the comparative position of the three raw angles: the gaze–ball angle, head–ball angle, and gaze–head angle (Fig. 2). The purpose of calculating these angles was to quantify the relative movement of the eyes, head, and ball in relation to the player's gaze and head orientation. This provides insight into the visual strategies used by athletes with vision impairment during dynamic tasks such as returning serves. These angles were later aggregated to perform time-series analysis using dynamical time warping (DTW) (see Data Analysis).

To identify saccades, we first smoothed the gaze data using polynomial smoothing within a window of 0.02 seconds. This strategy allowed us to calculate the velocity and acceleration of the eyes at each moment. Velocity was computed by taking the second-order derivative of the polynomial fits to the horizontal and vertical gaze data, squaring these derivatives, and summing them. Acceleration was computed similarly by taking the third-order derivative of the polynomial fits, squaring these derivatives, and summing them. We then ranked these velocity and acceleration values and computed probabilities for each. The ranks were transformed into probabilities using a power law. This transformation was designed such that only the top 10% of the ranked velocities and accelerations have a probability of greater than 0.5. To identify saccades, we thresholded these probabilities: values of greater than 0.5 indicated potential saccades. We refined this identification by ensuring the orientation of the saccade was consistent in a straight line. To achieve this, we calculated the overall direction vector of the saccade by determining its horizontal and vertical components. We then computed the dot product between the segment vectors within the saccade and the overall direction vector. Any segment that deviated from the overall direction vector, as indicated by a negative dot product, was excluded from being classified as part of a saccade. Additionally, we ensured that consecutive saccades were separated by at least 50 ms. If two saccades were closer together, they were combined into one, unless they were in the same direction; in this case, the smaller of the two saccades was discarded. Finally, we required that the amplitude of the overall change in eye orientation during a saccade was at least 1°. Saccades that did not meet this criterion were discarded. This analysis was performed using a custom written Python code (Version 3.9) and was similar to the analysis in Ghiani et al. (2024).^34^

### Statistics

First, we had differences in the duration of our time series data within and between participants for at least three reasons. First, unlike many previous studies,^4^^,^^6^ ball speed was not standardized by using some sort of ball machine. In this study, we took a more ecologically valid approach by asking two tennis players to perform the serves, resulting in some degree of difference in the velocities of the serves. Second, participants were allowed a different number of bounces before being required to hit the ball (depending on their competition class), resulting in further differences in time between the serve and moment of return. Third, owing to different gaze and head strategies between participants, not all reference points were visible in the view of the head-mounted camera throughout the whole serve, and therefore the final moments of the return were necessarily cut short on some trials.

**Figure 3.**
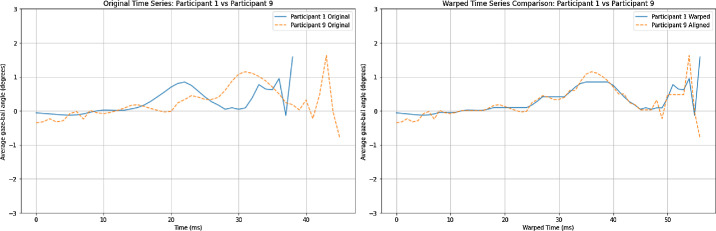
An example of DTW of the average gaze-ball angle time series to obtain the optimal alignment between the patterns of participants 1 and 9.

We used DTW to account for the differences in the time series data. DTW is robust to the difference in the length of time series by warping or stretching the time axis to determine the most optimal alignment between the two time-series (Fig. 3). Specifically, DTW was adopted to quantify, between each participant, the alignment of the participants' average gaze–ball time series during the duration of the serve (from server release to racket-ball contact). At its core, DTW seeks to find the optimal alignment between two time series that may vary in length or speed, allowing for the comparison of their similarities and differences.^35^^,^^36^ This is particularly valuable when dealing with temporal data where direct point-to-point matching is not feasible owing to differences in timing, rate, or duration. The algorithm works by dynamically warping or stretching the time axes of the time series to minimize the cumulative distance between corresponding points. By finding the most favorable alignment, DTW provides a measure of similarity and dissimilarity between the time series (Fig. 3).

A hierarchical clustering algorithm using the Ward method was applied to the resulting DTW distances to group the most similar gaze patterns.^37^^–^^39^ A DTW distance is a value that represents the cumulative difference between the aligned points of two time series after applying the DTW algorithm. In our hierarchical clustering analysis, we set the distance threshold for merging clusters to 250 units. This threshold is based on the DTW distance scale used to measure the similarity between time series. We examined how the number of clusters varied with different distance thresholds. By plotting the number of clusters against the distance threshold, we identified an elbow in the plot. This elbow point represents a considerable change in the number of clusters, indicating a suitable threshold where the addition of more clusters yields diminishing returns. The threshold of 250 was chosen because it provided a balance between having a manageable number of clusters and maintaining meaningful groupings.

All statistical analyses were performed using a custom written Python script (version 3.11). Alpha was set at 0.05 for all comparisons.

## Results

In this study, we recruited 19 participants initially; however, we were unable to succesfully calibrate the eye tracker for 8 participants and so their data were excluded from the analysis (Table). The challenges with calibration were associated predominantly with ocular conditions affecting the anterior eye segment (e.g., aniridia, coloboma) or nystagmus (uncontrollable shaking movements of the eye). The final sample comprised 11 participants (5 females) with an average age of 46.3 years (range, 29–57 years) and an average tennis experience of 10.2 years (range, 4–20 years) (Table).

**Table.** Participant Characteristics

Participant	Age (Years)	Tennis Experience (Years)	Competition Class	Ocular Condition	Eye Tracker Calibration	Number of Analysed Trials
1	44	8	B3	Retinitis Pigmentosa	Yes	40 (100 %)
2	65	8	B3	Nystagmus	No	0
3	52	20	B3	Congenital Rubella	Yes	40 (100 %)
4		15	B3	Toxoplasmosis	No	0
5	62	12	B2	Aniridia, cataract	No	0
6	31	10	B4	Astigmatism, Nystagmus	Yes	39 (97.5 %)
7	40	11	B2	Coloboma	No	0
8	66	8	B3	Ocular albinism	No	0
9	29	5	B4	Retinitis pigmentosa	Yes	33 (82.5 %)
10	57	9	B2	Macular degeneration	Yes	40 (100 %)
11	48	10	B3	Congenital cataract	Yes	40 (100 %)
12		1	No classification	Cone dystrophy	No	0
13	59	15	B2	Stargardt's disease	Yes	39 (97.5 %)
14	39	6	B2	Stargardt's disease	Yes	39 (97.5 %)
15	54	9	B3	Stargardt's disease, nystagmus	Yes	39 (97.5 %)
16	50	16	B2	Retinitis pigmentosa	Yes	40 (100 %)
17		4	B3	Stargardt's disease	Yes	39 (97.5 %)
18	16	4	B4	Marfan's syndrome	No	0
19	36	10	B2	Glaucoma, cataract	No	0

**Figure 4.**
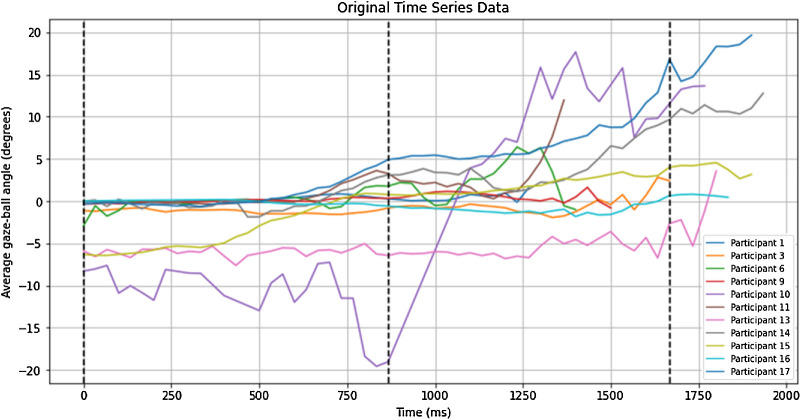
Time series data for the gaze-ball angle in degrees over time for each participant. The first vertical dotted line represents the moment of racket-ball contact for the server, the second line the median timing of the first bounce, and third line the median timing of the second bounce (for some participants).

The participants showed an excellent ability to return tennis serves, with a median return percentage (number of returned serves/total number of serves × 100) of 97.5% (IQR, 97.5%–100.0%). There was a large variety in the gaze strategies used when returning the serves, particularly around the moment(s) of ball bounce, as shown in Figure 4.

**Figure 5.**
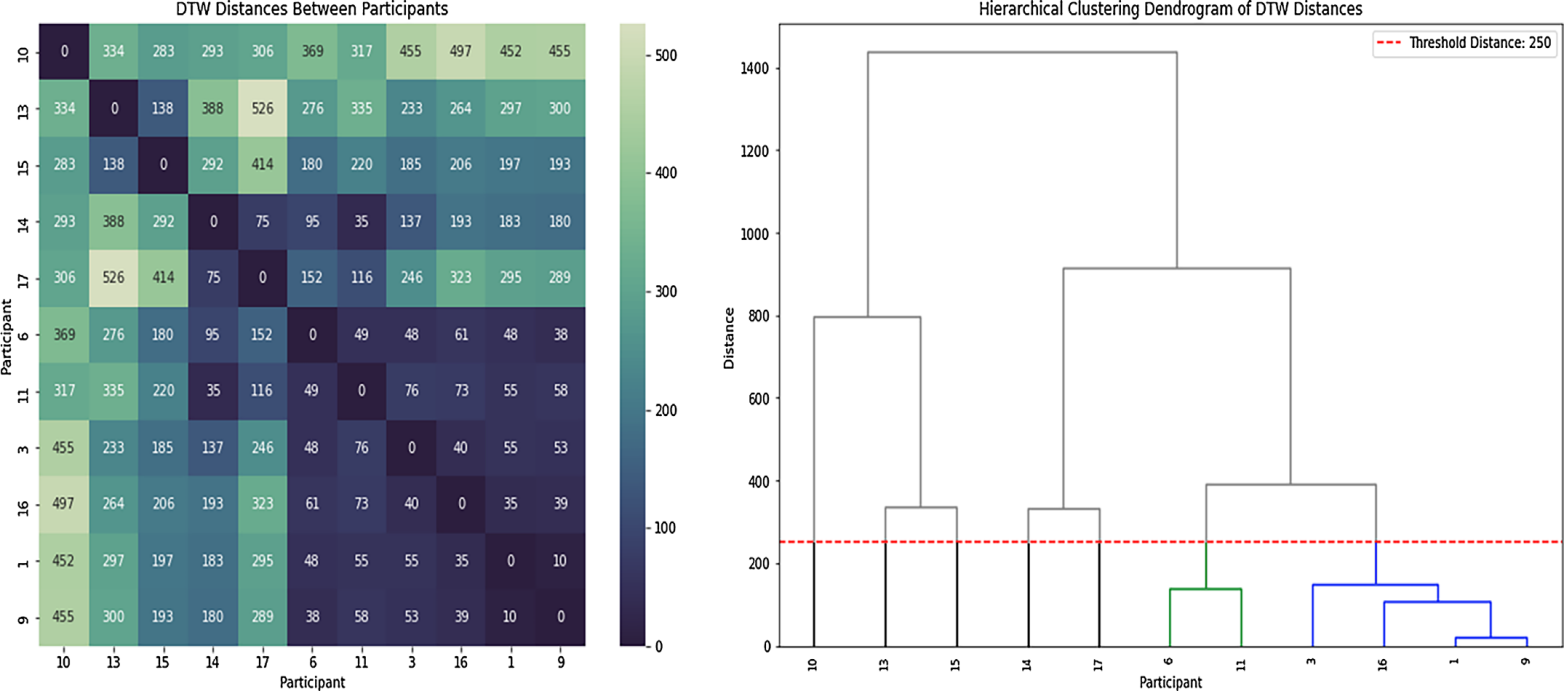
Clustering of participants based on DTW distances between time series of average vertical gaze-ball angles. Left: similarity matrix resulting from the DTW analysis, lower scores indicate greater similarity between the participants. Right: Results from the hierarchical clustering of the DTW distances. Colors indicate the emergent clusters, in black the unclustered participants, using a cutoff distance of 250 units.

DTW was conducted to compare the gaze time series data pairwise between participants. The DTW of the time series for each participant's average vertical gaze–ball angle was performed to determine how much the time series aligned to each another regardless of the length of the time series or the ball speed, with lower values indicating greater alignment (Fig. 5 left). Subsequent hierarchical cluster analysis on the DTW distances revealed which participants had the most similar patterns and grouped those into clusters (Fig. 5 right). Based on the chosen DTW distance of 250 units, two clusters emerged, in addition to a group of 5 individual participants who did not fit into a distinct cluster (Fig. 5 right). Cluster 1, which we labelled as the *peripheral vision-loss* cluster, had the greatest alignment between participants (lower right in Fig. 5 left, far right in Fig. 5 right). The peripheral vision-loss cluster consisted of participant 1 (condition: retinitis pigmentosa; class: B3), participant 3 (condition: congenital rubella; class: B3), participant 9 (condition: retinitis pigmentosa; class: B4), and participant 16 (condition: retinitis pigmentosa; class: B2). Cluster 2, which we termed the *ocular misalignment* cluster, consisted of participant 6 (conditions: astigmatism and nystagmus; class: B4) and participant 11 (congenital cataract; class: B3). The remaining five participants that were not clustered together, who will be referred to as the *stand-alone participants*, were participant 10 (condition: macular degeneration; class: B2), participant 13 (condition: Stargardt's disease; class: B2), participant 14 (condition: Stargardt's disease; class: B2), participant 15 (condition: Stargardt's disease; class: B3), and participant 17 (condition: Stargardt's disease; class: B3). Although not clustered together, it is noticeable that those participants were all characterized by central vision loss. Participants within each cluster differed in their sport class, suggesting that gaze patterns were not dictated by the severity of impairment.

**Figure 6.**
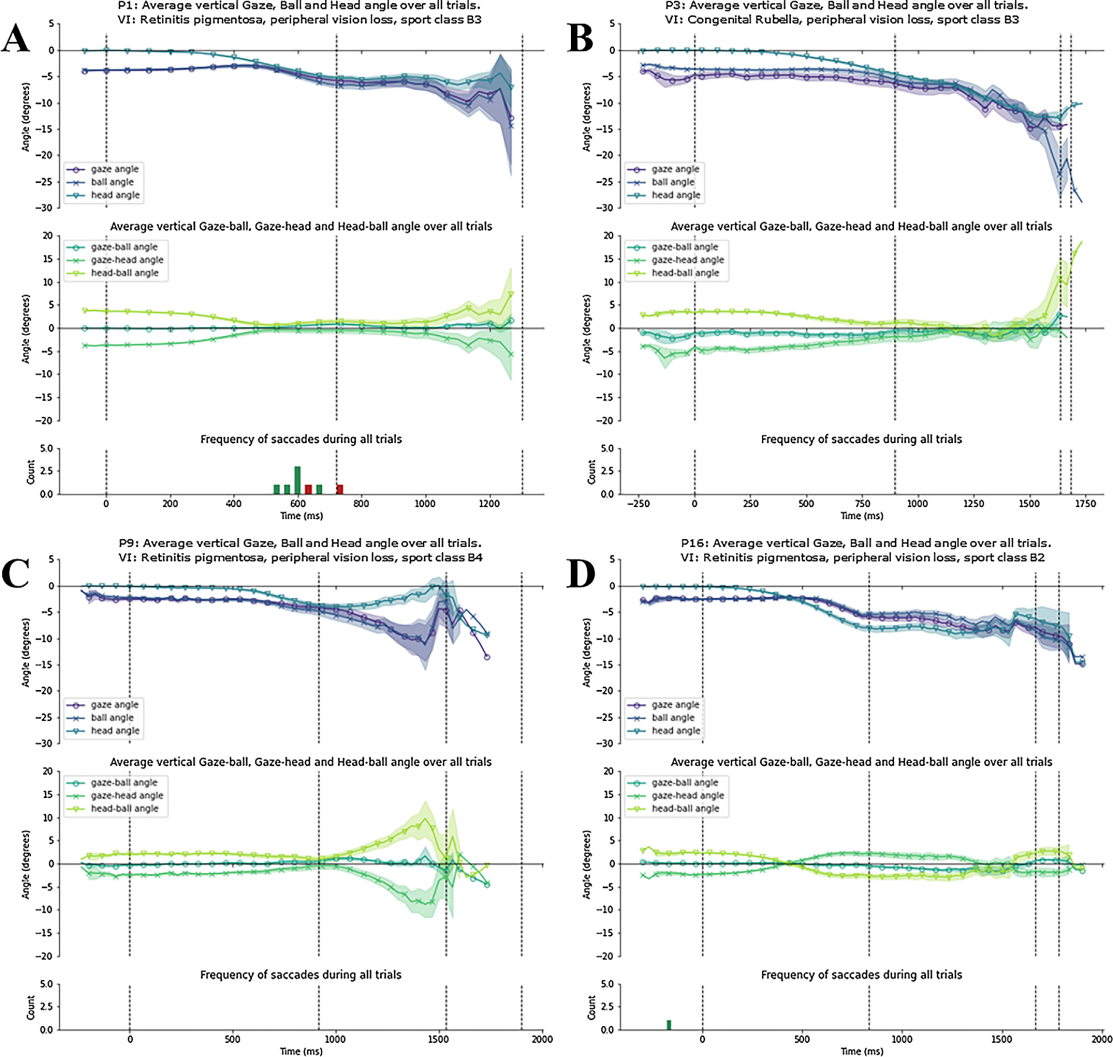
Gaze behavior of individual participants in the peripheral vison-loss cluster. (**A**–**D**) Data for individual participants (participants 1, 3, 9, and 16). In each panel, the top plot displays the average vertical gaze, ball, and head angle with SE in shading; the middle plot displays the average vertical gaze-ball, gaze-head, and head-ball angle with SE in shading; the bottom plot displays the frequency of saccades during the trials, with red bars indicating those saccades where gaze moved from above the ball to below the ball. The first and last dotted vertical line represents the average moment of racket-ball contact at the serve and racket-ball contact for the return, respectively; the dotted vertical lines in between represent average moments of ball bounce.

**Figure 7.**
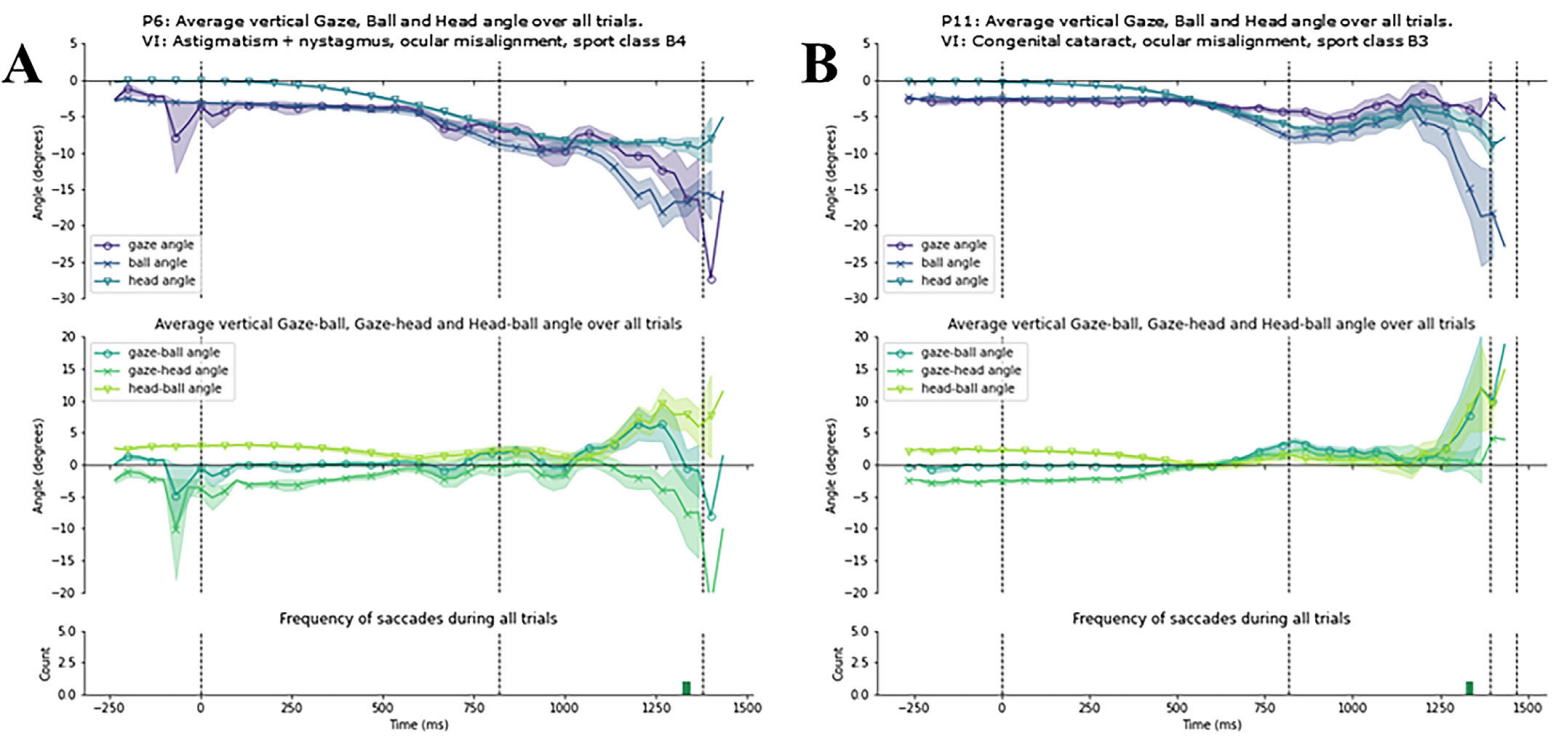
Gaze behavior of individual participants in the ocular misalignment cluster. (**A** and **B**) Data for individual participants (participants 6 and 11). In each panel, the top plot displays the average vertical gaze, ball, and head angle with SE in shading; the middle plot displays the average vertical gaze-ball, gaze-head, and head-ball angle with SE in shading; the bottom plot displays the frequency of saccades during the trials, with red bars indicating those saccades where gaze moved from above the ball to below the ball. The first and last dotted vertical line represents the average moment of racket-ball contact at the serve and racket-ball contact for the return, respectively; the dotted vertical lines in between represent average moments of ball bounce.

**Figure 8.**
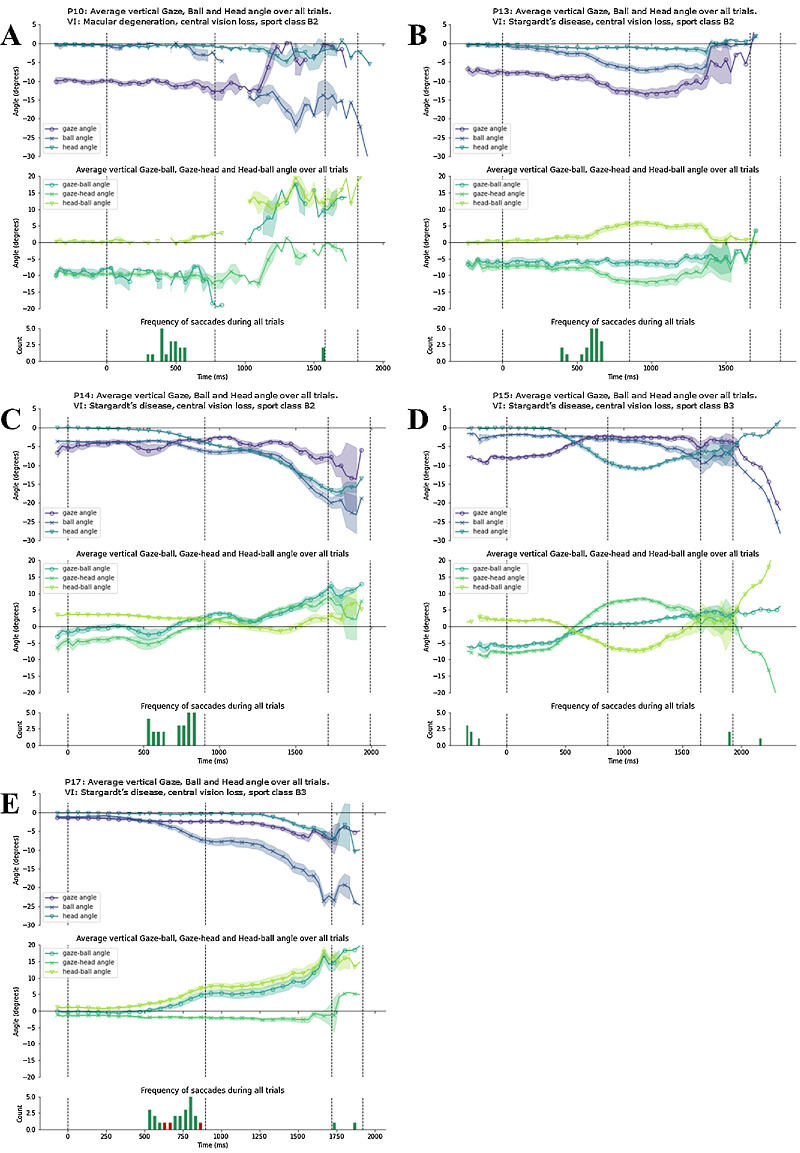
Gaze behavior of participants 10, 13, 14, 15, and 17 (**A**–**E**). In each panel, the top plot displays the average vertical gaze, ball, and head angle with SE in shading; the middle plot displays the average vertical gaze-ball, gaze-head, and head-ball angle with SE in shading; the bottom plot displays the frequency of saccades during the trials, with red bars indicating those saccades where gaze moved from above the ball to below the ball. The first and last dotted vertical line represents the average moment of racket-ball contact at the serve and racket-ball contact for the return, respectively; the dotted vertical lines in between represent average moments of ball bounce.

To examine the gaze patterns of each cluster, we present all individual time series data within each cluster. Figures 6A–D display for each participant in cluster 1 the vertical gaze, head, and ball angles over time (top); the vertical gaze–ball, gaze–head, and head–ball angles over time (panel); and the frequency of saccades across all trials (bottom). Figures 7A and 7B do the same for the participants in cluster 2, and Figures 8A–E for the stand-alone participants.

### Clusters

#### Cluster 1: Peripheral Vision Loss

Cluster 1 comprised participants 1, 3, 9, and 16. Three of the four participants in this cluster had the same ocular condition, retinitis pigmentosa (typically causing loss of peripheral vision), but with different levels of severity (i.e., they would not compete in the same class). The other participant had rubella syndrome (congenital viral infection interfering with normal development of the eye, which commonly manifests in congenital glaucoma or retinopathy). This group is characterized by very close tracking of the ball with gaze, and with very few saccades throughout ball flight. Notably, the participants with retinitis pigmentosa in this cluster coordinated the movements of their eyes and head to maintain near-perfect gaze alignment with the ball (Figs. 6A, 6C, and 6D). The remaining participant with rubella (participant 3) also displayed consistent ball tracking throughout ball flight, however, with the gaze directed slightly below the ball during its trajectory (Fig. 6B).

#### Cluster 2: Ocular Misalignment

Cluster 2 comprised participants 6 and 11 and is characterized by gaze progressively falling behind the ball as it approaches the participant. Participant 6 had nystagmus, strabismus (misalignment of the eyes), and astigmatism (refractive error), and participant 11 had congenital cataract (a condition that interferes with normal development of the eye, sometimes also resulting in strabismus and nystagmus). Both participants likely had ocular misalignment in common. The participants tracked the ball with their gaze throughout most of the ball-flight up to the moment of the first bounce, but from there gaze moved above/behind the ball in the lead up to and after the first bounce (Figs. 7A and 7B). Gaze realigned with the ball only once for a brief moment between the first bounce and contact or the second bounce.

### Standalone Participants

Participants 10, 13, 14, 15, and 17 had gaze patterns that were dissimilar to all other participants and to each other (Figs. 8A–E). Notably, each of these stand-alone participants had some form of macular loss (e.g., Stargardt's disease). Yet, despite the similar medical conditions, they each had quite distinct forms of gaze behavior to each other (as evidenced by the large DTW distances and inability to group together into one cluster).

Participant 10, diagnosed with severe macular degeneration, was characterized by a remarkable shift in the area of retina presumably used to track the ball (Fig. 8A). There was a considerable amount of missing data for participant 10, not necessarily owing to problems with the eye tracking per se, but rather owing to the substantial approximately 10° gaze offset below the ball at the moment the serve began. This offset meant that it was not possible to locate some of the locations in the scene camera footage required for the digitization of the angles at some moments during ball flight. As a result, there is some data loss, and this factor might at least in part explain the considerable variability seen in their gaze data. Nonetheless, the pattern suggests that participant 10 tracked the ball rather consistently using their superior visual field (inferior retinal locus) up to approximately 200 ms before the first ball bounce. Predictive saccades helped to move the gaze even further below the ball in the period immediately before the first ball bounce, with gaze almost directed toward the participant's feet at that moment (based on manual viewing of the video footage). There is a dramatic change in between the first and second bounce though, with the participant switching to use their inferior visual field (superior retinal locus) to view the ball for the remainder of its trajectory. As the ball moved downward after the first bounce, the eyes instead moved upward, by approximately 10°, meaning that the ball then remained approximately 10° *below* the direction of gaze. This dynamic switching of the peripheral location was not coupled with head movement: the head remained relatively still throughout the serve.

Participant 13, who had Stargardt's disease, seemed to use a more consistent area of their peripheral vision to track the ball throughout its ball flight (Fig. 8B). Their gaze was approximately 6° below the ball as the serve commenced, and they moved their gaze in a manner that ensured the ball remained consistently within that area of the superior visual field (i.e., a single inferior retinal locus). Although participant 13 did not switch the area of retina they used to track the ball, there were other similarities to the behavior of participant 10. First, participant 13’s head barely moved during the return of serve action, as indicated by the relatively consistent head angle throughout the ball's trajectory (Figs. 8A and 8B). This finding suggests that most changes in gaze position were achieved via eye movements rather than head movements. Second, participant 13 also used predictive saccades in the moments leading up to the first ball bounce to move gaze further below the ball in the lead up to the moment of bounce (Figs. 8A and 8B).

Participant 14, who had Stargardt's disease, dynamically moved their gaze below and above the ball, presumably using different parts of their retina to view the ball. Their gaze seemed to swerve up and down, starting with the gaze just below the ball, with short moments where gaze was aligned with the ball, and gaze ultimately ending up well above the ball toward the end of its trajectory (Fig. 8C). Gaze tracked the ball relatively well up until predictive saccades moved gaze below the ball after approximately 500 ms of ball flight. After that point, the eyes tended to remain rather stationary until late in the trajectory, with only a small amount of downward head movement, meaning that the angle between gaze and the ball grew almost linearly owing to the ball's downward movement in the visual field. It was only in the lead up to and moments following the second bounce that gaze started to move downward again, though by that point gaze was and remained approximately 10° above the ball.

Participant 15, who had Stargardt's disease, used a strategy characterized by a distinct shift in the area of retina used to track the ball. Their gaze behavior was very consistent across trials, as evidenced by small standard errors in Figure 8D. Initially, gaze was located about 5° below the ball. Gaze remained there for approximately 400 ms (about half-way between the start of serve and first ball bounce), at which the participant started moving their head downward, and their eyes upward to partially compensate. This meant that gaze was directed toward the ball immediately prior to the bounce and remained there for approximately 200 ms after bounce by virtue of the continuing downward head rotation. After that, the eyes remained still, but the head started to rotate upward, causing the ball to move into the inferior visual field. The ball then remained approximately 5° below gaze through to the moment of contact. As a result, participant 15 also altered the area of the retina used to view the ball. Similar to participant 10, participant 15 initially used the superior visual field (inferior retinal locus) to track the ball, then switched to a retinal location close, but superior to the macula, to continue tracking the ball with the inferior visual field (Fig. 8D). The shift in this case was achieved largely by a complex counter-rotation of the head and eyes up to bounce, and then by head movements after the first bounce.

Participant 17, also with Stargardt's disease, exhibited, remarkably, very little eye and head movement. This very stationary behavior is evident in the flat traces for the gaze, head, and gaze–head angles (Fig. 8E). There was a relatively small amount of head movement that started approximately half-way between the first and second ball bounce, and then two upward shifts in the eyes before and after the second bounce. Despite these late shifts, gaze was up to 20° above and behind the ball at the moment of racket-ball contact (and participant 17 did successfully return the ball on all but one trial). Saccades were also frequent immediately before the first bounce, though these were not particularly evident as changes in gaze–ball angle.

## Discussion

The aim of this study was to uncover the gaze patterns used by tennis players with vision impairment when hitting a moving ball. Usable eye-tracking data were collected for eleven competitive tennis players who each returned forty tennis serves hit by a live opponent. From these data we could derive the gaze, ball, and head angles, as well as the relative angles between each of those. Our results indicate that, despite high degrees of vision impairment, the participants were very successful in returning tennis serves, with most participants recording a (near) perfect percentage of returns. However, we found a large degree of variability in the gaze strategies used by participants when returning the serves. Cluster analysis on the similarity scores of the time warped time series revealed that the type of impairment shaped the participant's gaze patterns. Cluster 1 consisted mainly of those participants with peripheral vision loss, who displayed tight gaze–ball pursuit and very few predictive saccades. Cluster 2 consisted of two participants with some form of ocular misalignment, who tracked the ball initially but then fell further behind the ball as it approached them. A third group of stand-alone participants all had central vision loss, and could all hit the ball, yet they did so by each adopting their own unique gaze strategy that presumably differed in the peripheral location(s) with which the ball was being viewed. Through our analysis, we observed not only the commonalities (e.g., those with peripheral field loss), but also the individual idiosyncrasies (e.g., those with central field loss) that underpin the gaze behavior of individuals with vision impairment when performing a complex visual-motor task such as hitting a ball.

To our knowledge, this study was the first that investigated the gaze behavior of athletes with vision impairment during a sport. Our findings underscore the remarkable adaptability and skill demonstrated by individuals with vision impairment in meeting the visual demands of Para sports. Despite inherent challenges, such as severely reduced visual acuity, and/or central or peripheral field loss, the participants achieved an impressive median return percentage of 97.5%. Nearly one-half of the players managed to return all 40 serves. In this study, we were able to unravel the gaze patterns underlying the success of these tennis players with vision impairment.

Peripheral vision loss resulted in tight gaze–ball pursuit when tracking the ball. Participants in the peripheral vision loss cluster all used a very similar gaze strategy, typified by near perfect and consistent ball tracking throughout the return of serve, and an almost complete absence of the predictive saccades that are typically found when hitting a bouncing ball.^4^^–^^6^ Perhaps these findings should not be so surprising given that they are able to foveally track the ball, and that they would risk losing the ball if they were to move their gaze away from it (e.g., by producing predictive saccades). Therefore, the classic coaching advice to “keep your eyes on the ball” may actually be the most pertinent advice for players with peripheral vision loss.

The strategy associated with poor oculomotor control, observed in the two participants in cluster 2, initially involved good gaze–ball tracking, but with gaze falling behind the ball later in its trajectory. Ball tracking during the initial portion of ball flight is not particularly challenging, largely because the ball at this stage is looming in the air toward them, with very little in the way of vertical eye or head movements needed to track the ball. The gaze of the participants did however start to lag behind the ball from about the moment at which the ball started to move downward in their visual field. From that point onward, gaze typically remained a few degrees above the ball. This result suggests that the participants in this cluster may have lacked the ability to track the ball using smooth pursuit eye and/or head movements throughout the serve. The result aligns somewhat with similar findings from studies examining oculomotor behavior in related conditions. For instance, individuals with amblyopia, which sometimes also results in strabismus and nystagmus, exhibit delayed smooth pursuit and saccade initiation.^19^^,^^21^^,^^40^ This delay likely stems from deficits in motion perception and visual processing speed,^19^^,^^21^^,^^40^ resulting in a reduced ability to smoothly track a target.^40^ Similarly, tight gaze–ball pursuit might not have been feasible for the participants in the ocular misalignment cluster when compared with that seen in the participants with good central vision. The data from these gaze patterns can be applied in training interventions tailored to different types of vision impairment. For example, players with peripheral vision loss, who rely on tight gaze–ball pursuit, may benefit from exercises that enhance their smooth pursuit capabilities and peripheral awareness. Similarly, the identification of delayed gaze in players with poor oculomotor control suggests that targeted training to enhance visual tracking speed and coordination could improve their on-court performance.

Participants with central vision loss all had a common impairment, yet each used their own unique gaze strategy to compensate for that impairment. On the one hand, this variability might reflect the lack of an obvious strategy to use when central tracking of the ball is not possible. It seems logical that a tennis player with central vision loss should consistently use one area of their peripheral retina to track the ball, but evidently this might not be feasible and/or as easy as it appears. On the other hand, the variability in gaze strategies could stem from the diverse areas and sizes of central scotoma that can result from macular degeneration, which can differ not only between individuals, but also between eyes within the same individual.^13^ Consequently, individuals with macular degeneration often develop their own preferred retinal locus, or multiple loci.^13^ This results in each individual having a distinct location in the retina that they prefer to use, away from the fovea, with the magnitude and direction of the gaze offset dependent on the size and location of the scotoma. For example, an individual with a preferred retinal locus positioned toward the bottom right of the macula will also direct their gaze to the bottom-right of a target to see and attend to the target. Previous studies on the development of preferred retinal loci have shown that those who developed a well-defined retinal locus typically use a visual field location to the left or below the scotoma.^41^^,^^42^ The participants in our study also tended to initially use a preferred retinal location below the macula (gaze typically below the ball). However, most participants did not use that same retinal locus to track the ball throughout ball flight. Instead, four of the five of those participants with central vision loss seemed to systematically vary the area of retina they used to locate the ball. This result would not be expected if it were to be most beneficial to use a single retinal focus throughout ball flight. The result could be explained at least partly because smooth pursuit performance can be diminished with targets moving toward rather than away from the scotoma.^13^^,^^17^^,^^18^^,^^20^ Adaptations may be required because of an inability to track the target as it looms toward them.

A case study by Sullivan et al. (2008)^43^ previously demonstrated that an individual with central vision loss used multiple retinal loci to perform natural tasks such as making a sandwich or catching a ball. The chosen retinal locus is modulated by task demands, including the size and location of visual targets.^43^ In the case of returning a tennis ball, the ball is initially a small and relatively static (looming) target located at a considerable distance (court length, 18.2 m). However, during its trajectory, the size of the image of the ball on the retina increases, as does the vertical angle at the eye through which the ball travels. Therefore, the ball becomes a relatively larger and more dynamic target that may force some participants to switch to a more suitable peripheral location for the task at hand. Individual differences in the timing of retinal locus shifts are highlighted by participants 10 and 15, where a shift occurred before the first ball bounce for participant 15 and after the bounce for participant 10.

Sighted tennis players tend to move their gaze so that they track the ball throughout most of its path, with a predictive saccade forward toward the bounce, and then some variability after bounce, with some tracking, some predicting, and others falling behind.^6^^,^^16^ Here we see quite distinct behavior in the players with vision impairment. The strategies used by players with central vision loss differ in that they are driven by the necessity to rely on peripheral vision. Specifically, each individual seems to adapt their gaze strategy based on their unique visual constraints imposed by their central scotoma, making it difficult to follow a single, uniform strategy. Conversely, individuals with peripheral vision loss track the ball closely with central vision, more akin to what those without impairment do, except they seem to avoid the predictive eye movements forward that those without impairment typically rely on. Those with impairment do indeed appear to rely on different gaze strategies to those without.^3^^–^^6^ The range of different gaze strategies used to account for central vision loss suggests that, unlike sighted players who can rely on central vision for tracking, athletes with central vision loss must adopt highly individualized approaches. This finding highlights the importance of personalized coaching and training programs, because there is no one-size-fits-all solution for dealing with central vision loss in tennis. Although central pursuit of the ball seems preferable for optimizing return performance,^4^^,^^7^^,^^16^ it is not strictly necessary for successful racket-ball contact. Overall, the diversity in gaze strategies highlights the adaptability and individual differences in how athletes with central vision loss compensate for their impairment during dynamic sports activities. In particular, it suggests that those individuals with central vision loss might not necessarily rely on a single retinal locus for tracking moving targets as might otherwise be assumed. In addition to its applications in (Para) sports, these insights into adapted gaze strategies can help low-vision professionals develop more tailored interventions that leverage the individual's residual vision, whether for orientation and mobility training, vocational rehabilitation, or daily living skills.

In our study, we used balls with ball bearings inside them because this is what is used regularly in the competition played by our participants with vision impairment. This adaptation in particular allows those with no or almost no residual vision (B1 players) to participate. In this sense, the sound may have aided the visual behavior of the higher impaired players more than others. In other words, the same study conducted with regular balls without sound might have resulted in poorer gaze behavior, particularly for those with greater impairment. We used the balls with sound given that this is the situation that the participants were most accustomed to. A similar study without the use of sound might result in somewhat different results, particularly if seeking to compare the gaze of players with different levels of impairment.

Eye tracking offers promising opportunities to enhance the performance of athletes with vision impairment in sports. By analyzing gaze patterns and identifying areas for improvement, athletes and coaches can make informed adjustments to their training regimens and game strategies. Furthermore, personalized training programs based on individual gaze patterns can help athletes with vision impairment to maximize their athletic potential and achieve higher levels of performance in their respective sports. However, for certain ocular conditions, calibration of the eye tracker proved very challenging, preventing the accurate recording of gaze for a considerable proportion of participants in our study. Accurate gaze estimation with an eye tracker often necessitates the clear identification of the eyes' landmarks. Infrared eye trackers, like the one used in our study, reflect infrared light from the cornea, resulting in reflections that are detected along with the iris through image processing, enabling determination of the eye's position. Unfortunately, we had to exclude eight participants owing to the eye tracker's inability to estimate their eye position. These participants shared conditions such as an abnormal shape of the iris (aniridia), pupil (coloboma), or cornea (Marfan's syndrome); cataracts (also secondary to other conditions like glaucoma and Marfan's syndrome); or nystagmus (also secondary to conditions like ocular albinism). The challenges associated with these conditions underscore the limited research on eye tracking in individuals with such eye conditions.^44^ Rather than excluding participants based on potential eye tracking difficulties, it is crucial to inform the scientific field about the specific conditions and severities for which eye tracking was or was not feasible. New forms of eye tracking based on machine learning methods may offer promise as a means of better collecting data in individuals with impairment.^44^ Moreover, the higher sampling rates of the newer systems (e.g., upward of 200Hz) would help provide more insights into saccade dynamics (e.g., velocities, acceleration) and into the occurrence of corrective saccades. Such advancements could ultimately lead to sport-specific eye-tracking systems that provide real-time feedback on gaze performance, supporting both competitive athletes and their coaches in refining gaze strategies even in the presence of severe impairments.

Our study is not without limitations. First, we have presented a somewhat biased subset of medical conditions that cause vision impairment given the challenge we encountered in the calibration of the eye tracker for participants with certain ocular conditions. Although we were able to include people in particular with central or peripheral vision loss, we were less able to include participants with conditions such as aniridia, coloboma, Marfan's syndrome–related corneal abnormalities, cataracts, and nystagmus. Future eye-tracking research in individuals with vision impairment should consider using these new calibration-free eye trackers that will, first, need to be trained on individuals with vision impairment. Second, the results of our cluster analysis should be interpreted with caution because of the low number (*n* = 11) of participants. Although we categorized participants based on their ocular condition and resulting vision impairment, specific details, such as secondary conditions and other manifestations, as well as the exact severities of impairment, were often not available. This lack of comprehensive medical data limits the depth of our understanding regarding how specific aspects of vision impairment, beyond broad categories, may influence gaze patterns during hitting. Furthermore, the impact of serve depth was not considered in the analysis. Variation in the depth could conceivably alter the likelihood of a saccade to ball bounce or contact point occurring.^16^ Although we have no reason to believe that the serve depth did differ between participants, there is a possibility that some participants faced shallower or deeper serves, which could have influenced their gaze behavior and overall performance. Future research should take serve depth into account. Eye tracking can present significant challenges when used with people with vision impairment.^44^ In our study, we used a video-based mobile eye tracker given that we collected data on court when hitting actual tennis balls. An eye tracker of this nature required the manual digitization of the video footage rather than the use of algorithms to automatically analyze gaze as might be possible when performing a task on screen. The manual digitization does, of course, add a margin for error. Moreover, it required us to make assumptions about where gaze was directed when the system was calibrated, particularly for participants with central vision loss. In those cases, we assumed, based on previous experience, that the participants would be able to direct their central vision toward the calibration targets. It is possible that there was some error in doing so, and that their fovea was not precisely directed toward the target as might be expected in those with an intact fovea. Nonetheless, it is clear that the participants with central vision loss were largely tracking the ball with areas of their retina that were different to that used during calibration (i.e., the gaze angle is different to the ball angle) (Fig. 8). Moreover, any calibration offset does not change the central finding that the area of the retina used to view the ball in central vision loss changes in four out of five of the participants with central vision loss.

In conclusion, our analysis sheds light on the nuanced gaze behaviors of individuals with vision impairment during the challenging task of returning a tennis serve. The participants in our study demonstrated remarkable success in returning tennis serves despite often high levels of vision impairment. Our findings indicate that the type of vision impairment is more likely to dictate the type of gaze pattern used rather than the severity of the impairment. Through cluster analysis, distinct gaze patterns emerged, revealing both commonalities and individual variations among participants. Notably, participants with peripheral visual field loss exhibited consistent ball pursuit, whereas those with central field loss demonstrated a range of unique gaze behaviors. These insights not only enhance our understanding of the visual strategies adopted in sports for individuals with vision impairment, but also underscore the importance of personalized approaches in training and rehabilitation programs. Moreover, this study also opens the door for innovative ways to study how individuals with vision impairment use their remaining vision to overcome the inherent constraints posed by challenging motor tasks in sports.
